# Editorial: Dissecting antinutrient traits using omics approaches

**DOI:** 10.3389/fpls.2023.1234245

**Published:** 2023-07-11

**Authors:** Mehanathan Muthamilarasan, Manoj Prasad

**Affiliations:** ^1^ Department of Plant Sciences, School of Life Sciences, University of Hyderabad, Hyderabad, Telangana, India; ^2^ National Institute of Plant Genome Research, New Delhi, India

**Keywords:** nutrition, antinutrient components, antinutrient biosynthesis, grains, omics

The human population predominantly depends on plants and plant-based products for their food and nutrition. While plants are a major source of nutrients and health-promoting compounds, they also possess antinutrient factors that affect health and well-being. Global research focuses on improving important traits in plants, particularly food and nutritional security. However, few research groups work towards identifying and characterizing the antinutrient factors in food grains. These factors include cyanogen and cyanogenic glucosides, enzyme inhibitors, erucic acid, exorphins, goitrogens, lectins/hemoglutinins, lipoxygenases, nitrates, oxalate and oxalic acid, phytates, raffinose oligosaccharides, saponins, tannins, and other contextual antinutrients. Though these molecules are vital for the growth and development of plants, they pose health concerns to humans when consumed. In this context, this Research Topic was edited to collate the knowledge available on antinutrient research. The Research Topic received two research and three review articles. A comprehensive review of different antinutrients, their biosynthetic pathways, and physical and chemical methods to remove/reduce the antinutrients was provided by Duraiswamy et al.. The article provides an excellent timeline of research on this aspect and a compendium of genetic and genomic factors that underlie antinutrient biosynthesis. Also, the article provides a roadmap for using advanced genetic and genomic tools to reduce antinutrient content in food grains.

Jha et al. reviewed the importance of integrated breeding approaches to develop low antinutrient-containing legumes. Also, the article emphasizes improving the nutritional content of food legumes. The authors have enumerated the different dietary and antinutritional factors affecting human health and summarized the outcomes of research on biofortifying food legumes. The importance of genetic-, genomic-, small-RNA mediated- and genome editing-based strategies to achieve high nutrient and low antinutrient-containing seeds has been extensively discussed. Similarly, Sanyal et al. focused on a particular antinutrient, the raffinose family of oligosaccharides (RFOs). Though RFOs are carbohydrate reserves in grains, they have a negative effect on human health. While removing RFOs will affect the germination and growth of plants, strategies need to be devised to reduce the level of RFOs to not have an impact on germination and also have no effect on human health upon consumption. Thus, the authors suitably claim this as a ‘tightrope walk.’ In this direction, the authors have provided complete information on RFOs, including their structure, types, biosynthetic pathway, genetic control, and degradation and distribution of RFOs. Also, the authors have summarized the physiological implications of RFOs in plants and animals, followed by the antinutritional properties of RFOs. Most importantly, the review details the approaches for reducing RFOs by modulating the key genes in RFO biosynthetic pathway.

The exogenous application of Selenium (Se) in improving the nutritional content of mung bean was reported by Wang et al.. Two mung bean varieties treated with different Se concentrations were used to estimate protein, fat, total phenols, flavonoids, and phytic acid levels. The authors observed a significant increase in the vital nutritional components upon Se treatment, whereas there is no difference in phytic acid levels between Se-treated and control plants. Metabolite profiling using LC-MS/MS platform showed significant differences in Se-treated and control plants and identified unique metabolites in Se-enriched mung bean. LC-MS/MS analysis also suggested the importance of L-Alanyl-L-leucine, 9,10-Dihydroxy-12,13-epoxyoctadecanoic acid, and 1-caffeoylquinic acid as nutrient biomarkers for identifying nutrient-rich mung bean (Wang et al.). The potential use of microbes in reducing antinutritional factors was reported by Pham et al.. Through computational approach, the authors analyzed the genome sequence information of bacteria to identify the genes involved in the metabolism of antinutrient factors. The Four pan-genome analyses pinpointed the genes encoding for enzymes involved in antinutrient catabolism. The authors suggest using the bacterial cultures harboring these genes for effective antinutrient removal through fermentation and/or related processes.

In conclusion, the Research Topic provides insight into the ongoing research on identifying and characterizing antinutrients and devising appropriate strategies to reduce antinutrient factors. While most of the antinutrients have been identified, the research should focus on dissecting the pathways responsible for their biosynthesis and accumulation. Further, identifying genes/QTLs/alleles underlying antinutritional traits will enable the researchers to formulate appropriate strategies to reduce the antinutrient content. Targeted editing of genes playing roles in antinutrient biosynthesis is one of the approaches that researchers follow to lower the antinutrient concentration in the seed grains. However, the challenge is to reduce the levels but not hamper the growth and development of plants due to the non-availability of these factors, which are essential to plants but antinutrients to humans.

## Author contributions

MM and MP planned the editorial. MM wrote and MP edited the editorial. All authors contributed to the editorial and approved the submitted version.

